# Atrial Fibrillation in Cardiac Amyloidosis: A Multicenter Experience Comparing Novel Oral Anticoagulants and Warfarin

**DOI:** 10.3390/jcdd13060259

**Published:** 2026-06-11

**Authors:** Hussein Abdul Nabi, Luke Dreher, Michael Liu, Soad Al Osta, Suganya A. Karikalan, Eiad Habib, Hicham Z. El Masry

**Affiliations:** Department of Cardiovascular Disease, Mayo Clinic Hospital, Phoenix, AZ 85054, USA

**Keywords:** cardiac amyloidosis, atrial fibrillation, anticoagulation, thromboembolic events

## Abstract

Background: AF in the setting of cardiac amyloidosis is associated with a high risk of TEs, irrespective of CHA2DS2-VASc score. While warfarin has been the traditional anticoagulant, DOACs offer a promising alternative, but their safety in this population remains underexplored. This study aimed to evaluate the prevalence of thromboembolic events (TEs), including stroke and transient ischemic attack (TIA), and major bleeding events in patients with cardiac amyloidosis (CA) and atrial fibrillation (AF) treated with either warfarin or direct oral anticoagulants (DOACs). Additionally, we aimed to explore whether DOACs are at least as effective as warfarin in protecting against TEs in this population. Methods: This retrospective cohort study analyzed 422 patients with confirmed CA and AF from Mayo Clinic, with a median follow-up of 4.3 years. Data on anticoagulation therapy, baseline characteristics, and outcomes (TEs and bleeding) were collected. Statistical analyses included chi-square tests, *t*-tests, and Cox regression to assess the relationship between anticoagulation and TE. Results: Among 422 patients, 21 experienced a TE. The annual event rate was 0.83% for warfarin and 0.67% for DOACs, with no significant difference (HR 0.66, CI 0.22–2.01, *p* = 0.5). Patients with anticoagulation interruptions > 5 days had increased TE risk (HR 3.19, CI 0.97–10.5, *p* = 0.056). The bleeding rate was 9.9% over 4.3 years (2.33% per year), with no significant differences between anticoagulants. Conclusions: Both warfarin and DOACs have similar, low risks of TEs in CA and AF patients. However, anticoagulation interruptions were associated with increased TE risk, emphasizing the challenges in managing anticoagulation in this population.

## 1. Introduction

Atrial fibrillation (AF) is a common arrhythmia in patients with cardiac amyloidosis (CA) affecting up to two-thirds of patients with CA [1,2,3]. However, the prevalence of atrial arrhythmia differs depending on the type of amyloidosis, with transthyretin (ATTR) amyloidosis being more commonly associated with atrial arrhythmia compared to light-chain (AL) amyloidosis [2]. AF represents the most frequent supraventricular arrhythmia in ATTR-CA, with reported prevalence reaching up to approximately 88% in advanced disease [4]. Despite this high prevalence, AF is not consistently an independent prognostic factor in CA; however, its management remains clinically important for symptom control and quality of life, although it is often challenging in this population due to hemodynamic intolerance and comorbid disease burden [4].

Regardless of the amyloidosis type, patients with both CA and AF are at particularly increased risk of thromboembolic events (TEs) [5,6]. This can be multifactorial, not only related to atrial myopathy and decreased left atrial appendage emptying, but also to endothelial dysfunction [7,8]. Furthermore, left atrial function has been shown to be significantly impaired in patients with CA and is associated with an increased prevalence of AF [9]. Additionally, CA patients are at increased risk of bleeding secondary to amyloid angiopathy and other comorbidities, such as renal dysfunction, fall risk related to peripheral and autonomic neuropathy, and drug–drug interactions [6,10].

Due to the high risk of TEs, anticoagulation is recommended in all CA patients with AF, regardless of their CHA2DS2-VASc score (2023 ACC/AHA expert consensus) [11,12]. Traditionally, warfarin has been considered the anticoagulant of choice [10,12]. To further highlight the increased tendency for thrombus formation, it has been shown that the most common reason for canceling a direct current cardioversion for atrial arrhythmias in CA patients is intracardiac thrombus (27%), even in those adequately anticoagulated [13]. In a study of 116 autopsies, 33% of CA patients demonstrated an intracardiac thrombus. Patients with AL amyloidosis and AF are at an extremely high risk of TEs (OR 55, 95% 8.1–1131) [5].

Although the effectiveness of direct oral anticoagulants (DOACs) in reducing thromboembolic risk in patients with both CA and AF has not been extensively studied [6], DOACs offer the advantage of ease of use, without the need for serial monitoring of the international normalized ratio (INR), and lower risk of drug–drug interaction [14]. Therefore, the primary question addressed in this study is whether DOACs are as effective as warfarin in reducing thromboembolic events without increasing the bleeding risk in patients with both CA and AF.

## 2. Methods

### 2.1. Study Population

This retrospective cohort study analyzed patients with confirmed diagnoses of both CA and AF across Mayo Clinic sites in Rochester, Arizona, and Florida from 1992 to 2022. A database which includes demographic information, clinical characteristics, and comorbid conditions was built through the Mayo Clinic Data Explorer tool, with chart reviews conducted to verify the accuracy of the data entries and confirm the diagnoses. Informed consent was obtained from each patient. The study was approved by the Institutional Review Board (IRB) before data collection began. CA was further classified into subtypes: AL amyloidosis, ATTR amyloidosis (wild type vs. hereditary), or other forms. Similarly, AF was categorized as paroxysmal, persistent, or permanent. In addition to detailed patient histories, information regarding anticoagulation therapy and left atrial appendage occlusion (LAAO) procedures was obtained through chart reviews. Available echocardiographic data were also reviewed, with particular attention to left atrial volume index (LAVI), given its well-established association with atrial fibrillation and thromboembolic risk. Thromboembolic events (TEs), including stroke (confirmed by computed tomography [CT] or magnetic resonance imaging [MRI]), transient ischemic attack (TIA), or peripheral embolism, were systematically documented. For each identified TE, chart review was conducted to determine whether the event occurred while the patient was on therapeutic anticoagulation or if it was associated with interruptions (e.g., sub-therapeutic INR or peri-procedural factors). Bleeding events were identified through retrospective chart review and were defined as clinically significant bleeding events, including gastrointestinal bleeding requiring blood transfusion, intracranial hemorrhage, subarachnoid hemorrhage, and severe non-traumatic subdural hemorrhage. Given the retrospective nature of the study, bleeding events were ascertained based on their clinical relevance and documentation within the medical record rather than through formal adjudication according to a standardized bleeding classification system.

### 2.2. Study Outcomes

The primary outcome was the occurrence of TEs, and the study aimed to compare the incidence of these events based on the type of anticoagulation therapy the patient was receiving, as well as whether they occurred during an interruption in anticoagulation. The secondary outcome focused on the incidence of bleeding events within this population.

### 2.3. Inclusion and Exclusion Criteria

The inclusion criteria for this study include a confirmed diagnosis of CA and AF between 1992 and 2022. CA was confirmed through either a Technetium Pyrophosphate Scintigraphy (PYP) scan or a biopsy diagnosis of cardiac amyloid. Patients diagnosed with CA via PYP scan had a hematologic evaluation to rule out AL amyloid. For biopsy-confirmed cases, it was in the form of fat pad and/or bone marrow biopsy for diagnosis of AL amyloid, or endomyocardial biopsy for other forms of CA. For the analysis of TEs, including stroke and TIA, patients who were never anticoagulated at any point were excluded from the analysis. It is important to note that this exclusion differs from the interruption of anticoagulation, as, in our study, anticoagulation interruption was defined as a period lasting more than 5 days. However, patients who were not anticoagulated were not excluded from the bleeding analysis and were included in the study population.

### 2.4. Statistical Analysis of Thromboembolic Events

Categorical variables were reported as frequencies and percentages (*n*, %), while continuous variables were reported as mean and standard deviation (SD). The date where the anticoagulation was started is considered the index date, with patients right-censored at the date of their last follow-up. A Cox regression model was used to test the association between anticoagulation and TEs, treating anticoagulation as a time-dependent covariate. This approach was used to account for the possibility of patients receiving multiple anticoagulants (or no anticoagulant) during the study period. Events occurring within five days of anticoagulation discontinuation were attributed to the last anticoagulant regimen. A *p*-value ≤ 0.05 was considered statistically significant. Statistical analyses were conducted in R Statistical Software version 4.1.2.

### 2.5. Statistical Analysis of Bleeding Events

Statistical Package for the Social Sciences (SPSS) version 29.0 was used to compare the baseline characteristics between patients who experienced bleeding and those who did not. It was also used to calculate the frequency and percentage of bleeding events over the median follow-up period and to determine the annual bleeding risk.

## 3. Results

### 3.1. Demographics and Clinical Characteristics

**Figure 1:**
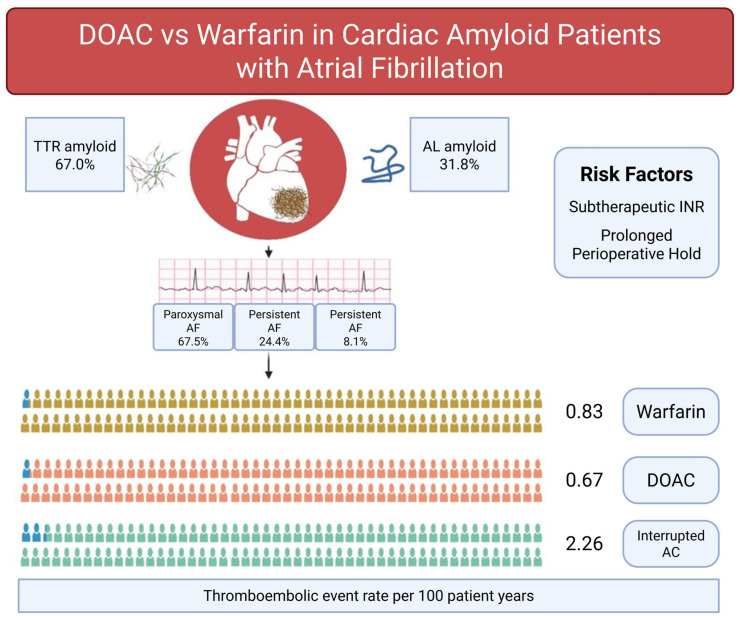
Central illustration. Abbreviations: AF: atrial fibrillation; TTR: transthyretin amyloidosis; AL: light-chain amyloidosis; INR: international normalized ratio.

This retrospective cohort study examined 422 patients diagnosed with both CA and AF who were prescribed oral anticoagulation therapy between 1992 and 2022. A detailed summary of the demographics and clinical characteristics of the cohort is presented in Table 1. The mean age of the patients was 71.7 ± 10.2 years, with a predominance of male patients (85.8%). The median follow-up duration was 4.3 years (95% CI: 3.9–4.7). Of the 422 patients, 282 (67%) had transthyretin amyloidosis (TTR-CA), and 134 (31.8%) had light-chain amyloidosis (AL-CA). Regarding AF classification, 285 (67.5%) had paroxysmal AF, 103 (24.4%) had persistent AF, and 34 (8.1%) had permanent AF. The median CHA2DS2-VASc score was 3.0 (IQR 2.0–4.0), suggesting a moderate-to-high risk of stroke within this cohort. The mean LAVI for the overall cohort was 45.09 ± 14.77. There was no significant difference in mean LAVI between patients receiving warfarin (45.77 ± 16.27) and those receiving DOACs (44.59 ± 13.85; *p* = 0.29). Figure 1 provides a summary of the key study results.

### 3.2. Thromboembolic Events and Survival Analysis

During the median follow-up period of 4.3 years, a total of 21 TEs occurred. Of these, 10 patients experienced an event while on warfarin over 1207.7 person-years (event rate: 0.83% per year). Six patients had an event while on DOACs over 897.1 person-years (event rate: 0.67% per year). Additionally, five patients experienced TEs while not receiving anticoagulation therapy for more than 5 days. Event rates stratified by anticoagulant type are outlined in Table 2.

No statistically significant difference in event rates was observed between patients on warfarin and those on DOACs [HR 0.66, CI (0.22–2.01), *p* = 0.5]. However, patients with interrupted anticoagulation therapy had a significantly higher event rate compared to those on continuous anticoagulation with warfarin [HR 3.19, CI (0.97–10.5), *p* = 0.056] (Table 3).

### 3.3. Clinical Characteristics of Thromboembolic Events

TE occurred in both amyloid subtypes, with 18 events observed among patients with ATTR-CA and three among patients with AL amyloidosis.

Among the 10 patients who experienced TE while on warfarin, nine had strokes, and one had a TIA. Of the nine stroke patients, five had sub-therapeutic INR levels (range: 1.2–1.6). After the event, seven patients continued warfarin therapy, two switched to DOACs, and one discontinued anticoagulation. In the six TEs occurred while patients were on DOACs, four were strokes, and two were TIAs. All six patients continued DOAC therapy, though in two cases, DOAC therapy was temporarily held for 6 days due to hernia and cystoscopy procedures. Table 4 provides a summary of the characteristics of patients with interrupted anticoagulation.

### 3.4. Bleeding Events

Among our patients with CA and AF, 55 experienced major bleeding events, irrespective of the anticoagulant used. The overall incidence of bleeding during a median follow-up of 4.3 years was 9.9%, translating to an annualized bleeding risk of approximately 2.3%. Among those patients, 30 experienced major gastrointestinal bleeding requiring blood transfusion, including 13 who were on warfarin versus 11 on DOACs, and six patients who were not on anticoagulation. Additionally, 25 patients had intracranial hemorrhages, which were categorized as subarachnoid, subdural, or intracerebral. Among these, 12 patients were on warfarin while 11 were on DOACs, and two patients were not on anticoagulation. There was no statistically significant difference in the bleeding rate between the two treatment groups (*p* = 0.186). It was notable that eight patients experienced major bleeding events while not on any anticoagulation.

## 4. Discussion

This study represents the largest cohort to date of patients with both CA and AF who received anticoagulation therapy. The overall rate of TEs was relatively low: 0.83 events per 100 patient-years for those on warfarin and 0.67 events per 100 patient-years for those on DOACs. These findings align with those from previous randomized controlled trials comparing DOACs and warfarin, which report similar event rates—ranging from 1.11% to 1.7% per year for DOACs and 1.5% to 2.2% per year for warfarin [15,16,17,18].

As expected, the rate of TE was notably higher among patients who experienced prolonged interruptions in anticoagulation (greater than 5 days). These patients had an event rate of 2.26 events per 100 patient-years [HR 3.19, CI (0.97–10.5), *p* = 0.056], emphasizing the increased thromboembolic risk associated with interruptions in anticoagulation therapy after a bleeding event or during the perioperative period.

In a previous study, patients with ATTR-CA reported higher event rates: 2.9 events per 100 patient-years for those on warfarin, 3.9 events per 100 patient-years for those on DOACs, and 1.9 events per 100 patient-years for those who were not receiving anticoagulation during the embolic event [14].

Many of the patients in this cohort had their anticoagulation temporarily withheld due to recurrent bleeding events, including hemorrhagic strokes and perioperative bleeding. This further complicates the interpretation of TE rates in this population.

Several factors likely contributed to the relatively low event rate observed in this cohort, including: (1) a higher proportion of patients with ATTR-CA (almost two-thirds of the cohort), (2) relatively fewer comorbidities, as reflected by a median CHA2DS2-VASc score of 3.0, and (3) a significant proportion of patients receiving disease-specific therapies [19].

Within the “on-treatment” group, an analysis of TE revealed that two patients on DOACs had their anticoagulation temporarily held perioperatively. Both experienced TE 6 days after the interruption, underscoring the heightened risk of thromboembolic complications in patients with CA and AF during perioperative periods. This suggests that bridging anticoagulation with parenteral agents during such interruptions may be necessary. Furthermore, among the 10 patients with events on warfarin, five had sub-therapeutic INR levels, suggesting that while warfarin can be highly effective when therapeutic, its variability in maintaining the correct INR range may make DOACs a more reliable option for these patients [14,20].

In this study, 55 patients experienced major bleeding events, resulting in an overall incidence of 9.9% over a median follow-up of 4.3 years, which translates to an annualized bleeding risk of approximately 2.3%. This is consistent with prior research, which highlights the increased bleeding risk observed in amyloidosis patients, particularly due to vascular fragility and amyloid deposition in the blood vessels, which can lead to spontaneous bleeding events [21]. In addition, microvascular endothelial dysfunction and vascular amyloid infiltration may further contribute to the complex balance between thrombosis and bleeding in this population [7]. Although the pharmacokinetic properties of DOACs might differ from those of warfarin, the extent to which these characteristics interact with amyloid-related microvascular abnormalities remains incompletely understood and warrants further investigation. In our cohort, gastrointestinal bleeding was the most frequent major bleeding event, followed by intracranial hemorrhage. This finding is aligned with previous reports that have highlighted the diversity of bleeding complications observed in amyloidosis patients, including both gastrointestinal and central nervous system hemorrhages [22]. These events occurred across patients with varying anticoagulation statuses: 22 patients on DOACs, 25 on warfarin, and eight not on anticoagulation experienced major gastrointestinal bleeding. This suggests that while anticoagulation therapy is an established risk factor for bleeding, it does not fully account for the observed bleeding complications in amyloidosis patients, which may be driven more by the underlying disease pathology itself, such as amyloid deposition in the vasculature [23].

The role of LAAO devices in reducing thromboembolic risk in patients with both AF and CA have not been evaluated in our cohort due to the small number of patients with CA and AF who underwent LAAO procedures. For patients at high risk for bleeding, the potential benefit of LAAO to reduce thromboembolic risk or mitigate stroke severity remains uncertain and warrants further investigation [7,24,25]. This protective role might be particularly important around the time of anticoagulation interruption perioperatively or after a bleeding event.

### Limitations

This study has several limitations. Firstly, it was a retrospective analysis conducted across three separate centers within a single institution at a tertiary referral center, whose practice patterns may differ from those at other clinical settings. This could limit the generalizability of the findings to broader populations. Secondly, the lack of consistency in anticoagulant use and in the duration of anticoagulant interruptions may introduce variability in the thromboembolic event rates. However, given that the interruptions were relatively brief in comparison to the total time patients were on anticoagulation, we believe that the event rate presented is a reasonable reflection of the actual risk. Thirdly, as an observational study, there is an inherent potential for selection bias, and randomized controlled trials are needed to provide more robust comparisons between DOACs and warfarin in this specific patient population.

Finally, the relatively small number of TEs limit the statistical power of the study and increases the possibility of a type II error when comparing anticoagulation strategies. The low event count also precluded more comprehensive adjusted and subgroup analyses, thereby limiting our ability to fully account for potential confounding factors. Consequently, residual confounding cannot be excluded, and the observed associations should be interpreted with caution.

## 5. Conclusions

In patients with both AF and CA, anticoagulation was associated with low rates of thromboembolic events, with warfarin and DOACs demonstrating similar efficacy and safety profiles. Due to challenges in maintaining stable INR levels and fewer drug–drug interactions, DOACs may offer practical advantages in this population. A proportion of thromboembolic events occurred during perioperative interruptions or periods of sub-therapeutic anticoagulation, suggesting a potential association between interruptions and thromboembolic risk. However, the observed association between interruptions >5 days and thromboembolic events did not reach conventional statistical significance (HR 3.19, *p* = 0.056). Given the high incidence of bleeding events in this population, careful individualized assessment of the risk–benefit ratio is essential when initiating or continuing anticoagulation.

## Figures and Tables

**Table 1:** Baseline characteristics.

Baseline Characteristics	*n* = 422
Age at initiation of anticoagulants, mean (SD)	71.7 (10.2)
Male	362 (85.8%)
**Type of Amyloidosis**	
TTR	282 (67.0%)
AL	134 (31.8%)
Other/unspecified	6 (1.2%) *
**Type of atrial fibrillation**	
Paroxysmal	285 (67.5%)
Persistent	103 (24.4%)
Permanent	34 (8.1%)
**Smoking status**	
Former smoker	176 (42.0%)
Current smoker	7 (1.7%)
Never smoked	236 (56.3%)
**Type 2 Diabetes Mellitus**	63 (14.9%)
**Hypertension**	241 (57.2%)
**CHA2DS2-VASc Score, Median (IQR)**	3.0 (2.0, 4.0)

* 1 patient with Apolipoprotein A IV type amyloid, 2 patients with AA amyloid, 1 patient with both TTR and AL, 1 patient with undifferentiated amyloid on cardiac MR, but elected not to undergo additional testing, 1 patient with unspecified type. Abbreviations: AL: light-chain amyloidosis; IQR: interquartile range; SD: standard deviation; TTR: transthyretin amyloidosis.

**Table 2:** Thromboembolic event rate by anticoagulant type.

Anti-Coagulant Type	Number of Patients *	Number of Events	Duration of Follow up (Person-Years)	Event Rate per Year	95% CI (for Event Rate)
Warfarin	228	10	1207.7	0.83%	0.40–1.5%
DOAC	303	6	893.0	0.67%	0.25–1.5%
Interrupted AC	79	5	177.1	2.26%	0.62–5.8%

Abbreviations: AC: anticoagulation; CI: confidence interval; DOAC: Novel Oral Anticoagulant. * Patients may fall under multiple anticoagulants or be represented multiple times for each anticoagulant due to changes in anticoagulation over time.

**Table 3:** Comparison of thromboembolic event rate based on Cox regression model *.

Anti-Coagulant Type	Hazard Ratio	95% CI	*p*-Value
Warfarin	-	-	-
DOAC	0.66	0.22, 2.01	0.5
Interrupted AC	3.19	0.97, 10.5	0.056

Abbreviations: CI: confidence interval, DOAC: Novel Oral Anticoagulant. * Excluding 2 patients with left atrial appendage occlusion device.

**Table 4:** Clinical characteristics of patients with a thromboembolic event.

Patient No#	Thromboembolic Event	INR at Time of Event	CHA2DS2-VASc	Sex	Type of CA	Age (Years)	Additional Comments
**Warfarin group**							
#1	Stroke	2.7	6	Male	TTR	82	a/c discontinued due to subarachnoid hemorrhage, considered for LAAO, but unfavorable anatomy
#2	Stroke	1.5	4	Male	TTR	84	Warfarin continued
#3	Stroke	1.6	7	Male	TTR	88	Warfarin continued
#4	Stroke	2.7	5	Female	TTR	90	Warfarin continued
#5	Stroke	1.4	4	Male	TTR	87	Switched to DOAC
#6	Stroke	1.2	3	Male	AL	59	Warfarin continued
#7	Stroke	Unknown	1	Male	TTR	74	Warfarin continued
#8	Stroke	2.9	3	Male	AL	66	Warfarin continued
#9	TIA	4.2	3	Male	TTR	72	Warfarin continued
#10	Stroke	1.7	1	Male	TTR	62	Switched from DOAC to warfarin (transplant listing) and had stroke, placed back on DOAC
**DOAC group**							
#11	Stroke	N/A	2	Male	TTR	81	DOAC continued
#12	TIA	N/A	5	Male	TTR	71	DOAC continued
#13	Stroke	N/A	3	Male	TTR	74	6 days hold for hernia repair DOAC continued
#14	Stroke	N/A	7	Male	TTR	81	6 days hold for cystoscopy DOAC continued
#15	Stroke	N/A	2	Male	TTR	87	DOAC switched (apixaban to rivaroxaban)
#16	TIA	N/A	4	Male	TTR	77	Patient also had LAAO DOAC continued
**No anticoagulation > 5 days**							
#17	Stroke	N/A	4	Male	TTR	73	Started DOAC
#18	Stroke	N/A	3	Male	TTR	74	DOAC started, LAAO then placed History of GI and cerebral amyloid angiopathy
#19	Stroke	N/A	5	Male	TTR	75	h/o bruising, never started on AC until after events, considering age started DOAC
#20	Stroke	N/A	0	Male	AL	68	Held warfarin for 11 days due to anemia and thrombocytopenia Resumed warfarin
#21	Stroke	N/A	3	Male	TTR	79	Held DOAC for 9 days due to subdural hematoma DOAC resumed

Abbreviations: AC: anticoagulation; CA: cardiac amyloidosis; DOAC: Novel Oral Anticoagulant; INR: international normalized ratio; LAAO: left atrial appendage occlusion; TIA: transient ischemic attack; TTR: transthyretin amyloidosis; N/A: Not available.

## Data Availability

The data underlying this article will be shared on reasonable request to the corresponding author.
